# Exploring the social brain

**DOI:** 10.7554/eLife.35392

**Published:** 2018-03-01

**Authors:** John P Welsh, Annette M Estes

**Affiliations:** 1 Autism CenterUniversity of WashingtonSeattleUnited States; 2 Department of PediatricsUniversity of WashingtonSeattleUnited States; 3 Center for Integrative Brain ResearchSeattle Children’s Research InstituteSeattleUnited States; 4 Department of Speech and Hearing SciencesUniversity of WashingtonSeattleUnited States

**Keywords:** EEG, autism, brain waves, social brain, brain connectivity, toddlers, Human

## Abstract

How does the brain physiology of young children with autism differ from that of typically-developing children?

**Related research article** Sperdin HF, Coito A, Kojovic N, Rihs TA, Jan RK, Franchini M, Plomp G, Vulliemoz S, Eliez S, Michel CM, Schaer M. 2018. Early alterations of social brain networks in young children with autism. *eLife* **7**:e31670. doi: 10.7554/eLife.31670

Children diagnosed with autism spectrum disorder (autism) process social cues atypically during the first year of life, and this sets them on a trajectory of increasingly impaired social ability. Understanding why this impairment emerges could help early intervention by identifying new intervention targets and the timing of intervention. Inherited genetic predispositions and epigenetic changes are often considered responsible for causing autism. However, solutions have not been readily forthcoming from that approach, largely because autism is caused by a complex interplay of alterations in cellular function, the electrical properties of neurons and their connectivity, and the activity of many different circuits in the brain. Since the behavioral symptoms of autism are expressed through brain physiology, interventions based on understanding and modifying brain physiology may be the best way to help affected children overcome their unique social challenges.

Now, in eLife, Holger Sperdin and Ana Coito, both from the University of Geneva, and colleagues report that they have compared, at the level of whole-brain neurophysiology, how three-year-old children with and without autism respond to social cues (Sperdin et al., 2018). Using multichannel electroencephalography (EEG), Sperdin et al. measured the flow of information in the cerebral cortex of the two groups as they watched the same videos depicting social stimuli.

When masses of neurons receive inputs together, they can create brainwaves – electrical signals that can be picked up through EEG (Olejniczak, 2006). Different brainwaves have different rhythms which can reflect various types of information (Lindsley, 1936; Grastyan et al., 1959). For example, theta rhythms (which oscillate between 4 and 7 times per second) signal cognitive workload and memory encoding, while alpha rhythms (8–12 times per second) often relate to mental relaxation and the inhibition of neuronal processing. Sperdin et al. used more than 100 EEG electrodes and a statistical approach called Granger-causal modeling to localize brain waves to specific brain areas.

The first notable finding was that information flowed very differently in the brains of children with autism during social processing. The changes in information flow were often driven by an increase in rhythmic activity within certain circuit nodes; these can now be identified as having a role in analyzing social information. The changes occurred for both the alpha and theta rhythms, and in nodes and pathways that were specific for each rhythm.

The second notable finding was that, in children with autism, not all the changes in information flow were maladaptive. Certain differences were associated with relatively normal visual exploration of social cues, and less clinical impairment in social behavior. Although the activity of neurons and circuits may be altered by changes that have a genetic origin, these results show that the brain can still compensate by deploying physiological adaptations to improve social performance. For instance, modifications in the amplitude and frequency of the theta and alpha rhythms can regulate perception levels (Linkenkaer-Hansen et al., 2004; Palva et al., 2005). Some of the changes observed by Sperdin et al. may therefore heighten perception levels and improve the ability of children to process social stimuli. If we can understand how the brains of children with autism compensate for deficits that they are born with, and why these mechanisms emerge, this could help to develop better behavioral interventions to guide social development at an early age.

A brainwave does not exist in isolation: it represents an important, but by no means final, stage of information processing. Large numbers of neurons may fire together due to the influence of synchronous synaptic input, but studying brainwaves will only tell us about the tempos and times of those influences. A key challenge for neuroscience is therefore to understand how brainwaves with different frequencies are integrated and thereby organize the firing of neuronal ensembles (O'Keefe and Recce, 1993; Buzsáki, 2006). In addition to providing a first look at how this process may be altered by autism, the findings of Sperdin et al. should inspire a new generation of integrative studies that examine how social behavior is regulated by the generation of electrical rhythms and pattern of electrical waves in the brain.
